# Personal perceptions of risky drinking and alcohol guidelines – a qualitative analysis

**DOI:** 10.1186/s12889-025-24296-6

**Published:** 2025-09-16

**Authors:** Joel Crawford, Richard Cooke, Gillian W. Shorter, Marcus Bendtsen

**Affiliations:** 1 Department of Health, Medicine and Caring Services, Division of Society and Health, Linköping University, Linköping, 581 83 Sweden; 2 School of Health, Education, Policing & Sciences, University of Staffordshire, Stoke- on-Trent, UK; 3 School of Psychology, Queens University Belfast, Belfast, UK

**Keywords:** Alcohol, Drinking guidelines, Risky drinking, Thematic analysis, Public health communication, Risk perceptions

## Abstract

**Introduction:**

Risky drinking is prevalent in European countries despite health authorities issuing guidelines created to support individuals to make informed choices about their alcohol intake. The current study explored personal perceptions of risky drinking, perceptions of the personal relevance of guidelines, and the processes used to form perceptions.

**Method:**

Three hundred and eight participants from Sweden and the UK completed an online survey containing open-ended questions about perceptions of risky drinking and personal relevance of drinking guidelines. Data was analysed using Thematic analysis embedded within a Framework analysis comparing answers from Sweden and UK.

**Results:**

Personal perceptions of risky drinking were formed primarily using experiential and affective judgments, and related to concerns over developing problems with alcohol, losing control, drinking as a coping mechanism, and causing harm to oneself or others. Guidelines were interpreted using experiential judgements, resulting in affective responses, expressed as negative attitudes towards the guidelines in terms of scepticism and denial. Those acknowledging the risk expressed ambivalence, in terms of a struggle for change.

**Conclusions:**

Perceptions of risky drinking extend beyond the physical act of consuming alcohol and encompass various contextual factors, affective states, effects of alcohol on the individual, and its impact on social roles and relationships. Risk guidance may not be interpreted as intended, with many who exceed the recommendations do not perceive their behaviour as risky. Understanding perceptions of risk can help support changes in behaviour,

**Supplementary Information:**

The online version contains supplementary material available at 10.1186/s12889-025-24296-6.

## Introduction

Globally, Europe is the continent with the highest per capita alcohol consumption [1]. In Northern European countries, like Sweden and the UK, around 80% of adults report consumption [1], with between 20 and 35% regularly engaging in heavy episodic drinking (i.e., consuming 60 g or more of pure alcohol in a single occasion) [1, 2]. Consuming alcohol in this fashion markedly increase the risk of experiencing chronic and acute consequences, including cancers, cardiovascular diseases, liver disease, accidents and injuries [3, 4]; this explains why Europe experiences the greatest burden of alcohol related harm globally [1] and the need for research into what causes adults in Sweden and the UK to engage in heavy episodic drinking.

Despite similar population prevalence of alcohol consumption, Sweden and the UK have different levels of alcohol governance; Sweden has a stricter policy compared to the UK (e.g., monopoly on sales and greater restrictions on advertising) [5]. However, health organisations/governments in both nations have used evidence from population risk models, that highlight a dose-response relationship between consumption and risk of harm [3] to create drinking guidelines, that typically advocating drinking within a limit of standard drinks, glasses, or units of alcohol [5–7]. In Sweden, adults are advised to drink less than 10 standard glasses per week (1 standard glass = 12 g of pure alcohol) and not exceed four standard glasses per session, more than once a month [5]. In the UK, adults are advised to drink less than 14 units of alcohol (1 unit = 8 g of pure alcohol) per week, and to spread drinking evenly across three or more days [6]. The UK guidelines were developed to help individuals make informed choices about their alcohol intake, whilst in Sweden the guidelines were developed to help identify individuals at risk, however, they have been promoted in the media as a way to make informed decisions [6, 8]. Despite widespread dissemination of the guidelines, 24% of adults in the UK and 40% in Sweden regularly exceed the respective limits and engage in drinking patterns that increase the risk of harm [9, 10].

National guidelines can provide guidance for individuals to moderate their consumption plus quantifiable measures for effectiveness trials [11], however, evidence highlights that most individuals are unaware of the guidelines and that they are perceived as having limited personal relevance for individuals who regularly consume alcohol [12–14]. One way to understand why messages are viewed as having limited personal relevance centres on how individuals perceive their own risks of experiencing alcohol harms.

Models of health (e.g., Protective Motivation Theory, Health Belief Model [15, 16]), define risk perceptions as subjective appraisals of (i) susceptibility (vulnerability) to a threat, such as the likelihood of experiencing negative health outcomes following alcohol consumption and (ii) the severity of a threat, such as experiencing acute or chronic harms following alcohol consumption. Health guidance on alcohol risk provides individuals with objective information regarding quantitative thresholds and subsequent risks of experiencing negative health outcomes. Processing this information requires that an individual engages in a deliberative judgement, in which a rational or logical evaluation of the guideline is performed [15, 16], e.g., understanding and interpreting the numeric information regarding the threat to health associated with a threshold amount of alcohol consumption. However, this type of risk communication is often difficult to interpret on an individual-level, as it may be misunderstood [17] or deemed as not personally relevant - personal intake often differs from the recommended amounts in the guidelines, in terms of computing units or standard drinks, remembering what was consumed, and the mathematical translation from bottles, pints, and shots [13, 14, 18, 19].

In contrast, evidence shows that individuals may use other means for assessing personal risk, such as experiential judgements, based on past outcomes or affective judgements, based on emotional reactions to a threat [20]. These subjective judgments can be driven by various factors that have been demonstrated to influence alcohol risk perception, including the experience of negative or positive consequences [21], drinking frequency and alcohol tolerance [22, 23], alcohol expectancies [24, 25] and normative beliefs [26, 27]. Evidence highlights that individuals may use these embodied means, based on their experiences for assessing personal risk, irrespective of public health guidance. This has been referred to as reaching the ‘tipping point’ (i.e., a subjective assessment of intoxication, whereby passing this level leads to impaired control and increases the risk of potential negative consequences) [28]. Furthermore, subjective ‘tipping points’ (i.e., personal alcohol limits) have been shown to far exceed guidance on single session drinking, and often are equivalent to, or surpass guidance on weekly drinking limits [29].

Risky drinking guidelines are an important part of the public health strategy for curbing alcohol-related harm, however the use of subjective experience for appraising personal risk and gauging limits suggests that health communication regarding risky drinking may require rethinking to convey its message in a way that is salient to the individual. To achieve this there is a need to understand how individuals perceive their personal risk and how they process current risk guidance (e.g., deliberatively, affectively or experientially). Whilst current understandings highlight potential experiential factors used to inform risk appraisal, less is known about how individuals conceptualise their own alcohol risk and the processes they use. Furthermore, interpretation of the current risk guidance at the individual-level has yet to be explored in Sweden and in the UK is limited to the previous guidelines^1^ [12].

The current study explored risky drinking perceptions in a sample of individuals who were seeking online support for changing alcohol behaviour as part of an RCT [30]. Exploring how treatment/support seeking individuals conceptualise risky drinking will increase the understanding of the perceived threat to health and help to inform intervention efforts at the individual-level (i.e., user-driven alcohol interventions), by providing insights into the psycho-social factors underlying behaviour change, such as barriers, beliefs and attitudes. Furthermore, exploring individual perceptions of the risky drinking guidelines will provide understanding of how alcohol policy translates to the individual and may provide insights that could be used to bridge the gap between policy and practice, whilst completing a cross-nation analysis between Sweden and the UK will enable a nuanced understanding of how or if differing alcohol policies influence individual perceptions. The current study explored how individuals from Sweden and the UK, who participated in a randomised controlled trial, perceive risky drinking and perceptions of personal relevance of the drinking guidelines.

## Method

The consolidated criteria for reporting qualitative research (COREQ) checklist was used to support study reporting [31] (see appendix A). Throughout the research process the lead author engaged with reflexivity, initially considering and noting how any preconceptions regarding the topic and how any past experiences with alcohol could influence the analysis, followed by how their preconceptions could impact the interpretation and writing.

### Participants and setting

Participants were recruited using online advertising (Google, Meta-Platform) to take part in a randomised controlled trial that estimated the effectiveness of drinking motives intervention content on drinking intentions, self-efficacy to reduce alcohol consumption, and reactivity to alcohol-cues [30]. The parent trial was registered prospectively on the ISRCTN registry on 06/03/24 (ISRCTN12456514), and the protocol is available online [30]. At the end of the trial, participants were invited to answer open-ended questions regarding their perceptions about risky drinking and drinking guidelines in their country. Inclusion criteria for the trial were (1) aged 18 or older and (2) consuming one standard Swedish drink (12 g of pure alcohol) in the last week or engaged in at least one heavy drinking episode (HED) in the previous month, defined as 48 g of alcohol in both locations, 4 or more standard Swedish drinks of alcohol or in the UK equating to roughly three 175 ml glasses of wine (2 units per glass = 16 g) or roughly three pints (568 ml) of beer (2 units per glass = 16 g).

### Data collection

A waiver for ethical approval was provided by the Swedish Ethical Review Authority on 2023-12-16 based on participants being anonymous (Dnr. 2023-06474-01). Prior to study commencement, participants read an information sheet, provided informed consent, and completed a baseline survey assessing demographics and alcohol consumption. They were advised that the study objective was to test how presentation of health information influenced their motivation to reduce alcohol consumption. After completing the experimental part of the trial, participants were invited to answer two open-ended questions; the first regarding their perception of risky drinking, “*In the box below*,* please describe your personal definition of risky drinking”*. After answering this question participants were presented with their relevant health authority’s definition of risky drinking (e.g., “*The National Board of Health and Welfare define risky drinking as consuming 10 standard glasses or more per week*,* or 4 standard glasses or more in a single session*” or “*The UK Chief Medical Officers defines risky drinking as consuming more than 14 units of alcohol per week”*, and a supporting image with the relevant drinks measures and then were asked, “*How relevant is this guideline to you?*” (see appendix B).

### Data analysis

Thematic analysis (TA), embedded in a framework analysis, was used to assess participants’ perceptions of risky drinking and personal relevance of the guidelines. TA is a qualitative technique that provides a means for producing a descriptive and informative assessment of a data corpus, that enables researchers to generate themes that conceptualise the meaning of a given phenomenon [32]. Framework analysis enables the comparison and contrast between two data corpuses, here between participants in Sweden and UK [33]. A separate analysis for each research question was completed for each sample, to avoid carryover effects from the interpretation of one phenomenon into another, in turn enabling a more focused analysis of how individuals frame their personal risk, along with how they perceive risk guidance.

The principal author led the analysis, in which they competed steps one to three outlined by Braun and Clarke [32] individually. This included (1) the familiarisation of the data set, which entailed repeated reading of the participants responses, along with making notes that expressed initial ideas. (2) Systematic coding, which entailed the use of words or statements to describe the sentiment of the text, completed iteratively in batches of 50 statements for the Swedish sample and 20 for the British sample until no new codes were found. (3) Generating of themes, assessing patterns and grouping of codes into overarching themes, using mapping. The principal and last author completed steps (4) in which they reviewed and refined the themes, assessing if they had clear patterns, were distinct and had enough support from the data excerpts and (5) defining and naming the themes, articulating what each theme is about by concisely conveying its scope and focus. Following the completion of step 5, a framework analysis was conducted. This entailed using a matrix to compare/contrast the themes identified from each sample. Finally, the principal author completed step 6, report writing supported by the other authors.

## Results

Out of the 554 individuals who participated in the trial, 308 (80 British and 228 Swedish) completed the open-ended questions. The item on personal perception of risky drinking, resulted responses ranging from 1 to 58 words, with an average of 8 words. For personal relevance of the guidelines, responses ranged from 1 to 66 words, with an average of 7 words. Table 1 highlights demographics and alcohol consumption for the total, British, and Swedish samples.

**Table 1:** Demographics and alcohol consumption of participants describing their risky drinking and personal perceptions of guidelines

	Total M (SD) or *n* (%)	UK sub-sample M (SD) or *n* (%)	Swedish sub-sample M (SD) or *n* (%)
Age	58.93 (11.41)	58.61 (12.15)	59.04 (11.17)
Gender			
Female	98 (32%)	22 (27.5%)	76 (33%)
Male	210 (68%)	58 (72.5%)	152 (67%)
Education level			
High school	38 (12%)	30 (37.5%)	8 (3.5%)
University	253 (82%)	42 (52:5%)	211 (92.5%)
Other	17 (6%)	8 (10%)	9 (4%)
Alcohol consumption			
Weekly consumption^a^	12.50 (12.28)	19.01 (14.72)	10.21 (10.41)
Monthly Heavy Episodic Drinking^b^	7.35 (8.42)	11:34 (10.41)	5.96 (7.11)

^a^Standard drink = 12 g of pure alcohol

^b^>4 standard drinks per session

### Perceptions of risky drinking

Results suggest perceptions of risky drinking are multifaceted: They include the physical act of consuming alcohol and the type of drinking, the context and the purpose of drinking, and how alcohol impacts cognitive processes, with personal health and social consequences central to perceptions. Participants here primarily used various experiential and affective judgements, along with some deliberative processing, often integrating the differing processes to inform their personal risk.

### Frequency and quantity

Regarding the physical act of drinking alcohol, a common theme identified was ‘Frequency and Quantity’. This theme highlights concerns over both the frequency and quantity of alcohol consumed, along with specific drinking patterns.

The theme suggests that how often and how much an individual drinks are key indicators of risky drinking. In forming this risk perception participants have used a mix of affective and deliberative judgements, derived from worries over developing problems with alcohol and their knowledge and/or beliefs regarding what constitutes problem drinking. For example, participants perceived a daily frequency of consumption, a pattern of drinking indicative of alcohol use disorders (AUDs) as a particular risk factor:

*“Drinking something alcoholic everyday”* – Female, 63, Sweden.

*“Drinking everyday”* – Male, 47, UK.

*“Having a daily relationship with alcohol*,* drinking every day even if it’s not much”* – Female, 25, Sweden.

Whilst both samples perceived concerns over the volume and frequency of drinking, such as daily drinking, the British sample expanded on this, by highlighting subjective thresholds for specific drinks (e.g., spirits) and how they are consumed as key indicators. Participant perceptions are likely formed from experiential and affective judgements, in which they have reflected on their subjective tolerance for certain drinks, and considered the negative outcomes from engaging with specific drinking styles. The potential for negative outcomes may give rise to an affective response, which heightens the sense of risk. This suggests certain types of stronger alcohol such as spirits, and methods of consuming that enables quicker absorption of alcohol into the bloodstream are indicative of increased risk:

*“Drinking the hard stuff*, e.g.,* 1 ltr Vodka in 2 days”* – Female, 62.

*“Drinking spirits in shots”* – Male, 75.

*“Drinking shorts as a chaser”* – Male, 65.

These risk perceptions may be driven by what the participants have felt or experienced from engaging with alcohol consumption, rather than what they know about alcohol and risk. However, when participants mentioned allied wording to the drinking guidelines in terms of frequency (weekly/daily) and quantity (units and drinks), their perceptions were higher than recommendations. This suggests that when individuals engage with deliberative judgements, informed by public health advice in terms of units or serving measures, a clear discrepancy between individual, subjective interpretations and national guidelines is evident. This disparity was seen in both countries:

*“More than 20 units a week”* - Female, 68, UK.

*“10 units or more per day”* - Male, 68, UK.

*“Over 2 glasses everyday”* - Female, 75, Sweden.

*“Drinking more than five drinks three times a week”* - Male, 29, Sweden.

### Losing control

A recurrent theme to define risky drinking related to an inability to control alcohol consumption. Participants highlighted that a loss of agency over control of consumption was indicative of risk. This perception is likely informed by experiential judgments based on participants’ direct engagement with alcohol consumption - here participants engaged with pattern recognition, consisting of a subjective assessment of the myopic effects of alcohol on their personal decision-making:

*“Not stopping when you’ve had enough”* – Male, 72, UK.

*“Not being able to have just one beer—it always turns into more”* – Male, 42, Sweden.

*“Not being able to stop when you want to”* – Female, 51, Sweden.

Further to the concern over losing control, both samples highlighted that extreme intoxication to the point of memory loss as indicative of risky drinking. These experiential judgements extend the pattern recognition process noted by other participants to include consequences to their antecedents, in this case negative outcome expectancies. This causal recognition process in which participants identify antecedents for behavioural patterns and subsequent outcomes highlights the experiential nature of individual-level risk appraisal. Furthermore, these perceptions suggest that extreme impairment from uncontrolled drinking may signal a more immediate and severe manifestation of risky drinking:

*“Blacking out”* – Female, 47, UK, “*Passing out*” – Male, 61, UK.

*“Drink so much that you don’t remember what you’ve done and can’t take care of yourself”* – Male, 67, Sweden.

“*Memory gaps*,* getting so drunk you lose control*” – Male, 29, Sweden.

The Swedish sample expanded on these sentiments by highlighting how the sense of losing control is further influenced by the myopic effect of alcohol on judgement, in which risky drinking is perceived as an inability to control both alcohol consumption and personal behaviour whilst under the influence. This perception may be informed by a mix of deliberative and experiential judgements, in which the participants have engaged with analytical reasoning based on experiential learning from the subjective experience of the cause-effect relationship of alcohol:

*“Drinking that exceeds the amount you have planned to drink*,* and that results in you doing things you would otherwise have refrained from”* – Female, 37.

*“If you can’t behave*,* talk normally or function normally. Not responding to speech and can’t decide for yourself when to stop drinking”* – Female, 44.

*“When you drink so much that you don’t care about the consequences and can’t control your intake. When you don’t reflect on your choices”* – Female, 40.

Furthermore, participants perceptions may be indirectly influenced by affective judgements, which are underpinned from the internalising and/or anticipating the potential fallout from failing to control themselves. For example, experiencing embarrassment, guilt, regret or shame after *‘doing things you would otherwise have refrained from’*. This highlights that personal risk perceptions for alcohol may be predicated on an interaction of cognitive, environmental and affective factors.

### Purpose

Across both data sets, perceptions of drinking for non-recreational purposes, like consuming alcohol alone and/or to relieve negative affect signified increased risk, suggesting that individuals’ using alcohol as a means of emotional or psychological escape could be risky. These perceptions highlight recognition of another form of drinking pattern, integrating affective judgement with experiential learning, to produce a reflexive assessment of personal risk:

*“Risky drinking means drinking too much and in the wrong contexts*,* such as drinking alone in depression*,* for anxiety relief*,* and out of boredom*, i.e.,* loneliness and social isolation”* - Male, 60, Sweden.

*“Binge drinking when sad and/or alone”* – Male, 28, UK.

*“Drinking to avoid thinking about certain things”* – Male, 66, Sweden.

Participants display interoceptive awareness of their affective reactions to stressful situations, and exposure to the outcomes of the maladaptive coping response enabled them to engage with reflexive thinking of how their behavioural patterns reinforce their sense of risk.

Further to this, other participants from both samples highlighted a more nuanced understanding of “*self-medicating*” (Male, 36, Sweden). This reflects both mental and physical challenges (e.g. experiencing insomnia and/or pain) and highlights how alcohol is used and to what end is a defining characteristic of risk as a coping mechanism. These participants also view risk through reflexive pattern recognition; risk is judged when alcohol becomes part of a negative, repeating cycle (e.g., alcohol is needed to sleep or relieve pain):

*“When you need it to cure pain”* - Female, 56, UK.

*“For pain in the joints”* – Male, 61. Sweden.

*“Drinking to sleep”* – Male, 66, UK.

*“More than three times a week to find peace and be able to sleep more than two/three hours a night”* – Male, 64, Sweden.

### Situational consequences

Both samples expressed situational consequences as characteristic of risky drinking. These perceptions of risk extend beyond the affective impact of alcohol to encompass how drinking negatively affects social roles, relationships, and safety. Participants’ notions of risk are potentially formed from experiential learning and subsequent affective responses. Direct experience of the consequences anchors the perception of risk, and the anticipation of negative affect, such as feeling guilt, regret or shame over their behaviour (or lack of action) amplifies the perceived severity and urgency of the consequences. This highlights risky drinking can be contextualised in terms of internal affective states and in the wider social environment:

*“Not fulfilling my commitments*,* the next day”* – Male, 50, Sweden.

*“When being drunk and causing offence to friends and family. When it effects your work”* – Male, 65, UK.

*“When you expose yourself or others to danger because of your intoxication”* – Male, 36, Sweden.

*“Drinking to the point where you harm yourself*,* your family and work”* - Male, 70, Sweden.

Further to this idea, other participants in both samples expanded on this theme by identifying situational contexts, such as drink spiking or drink-driving, that could render them in a vulnerable position. These perceptions again are based on experiential learning, with affective responses to the potential threat annealing the participants perceived vulnerability for experiencing consequences. This highlights that perceptions of risky drinking consider how impaired decision-making, risky behaviours, and context interact to elevate the sense of risk:

*“Leaving drinks unattended in a public place*,* accepting drinks from a stranger”* – Female, 54, UK

*“Not watching your drink in case*,* it gets spiked”* – Male, 64, UK.

*“Putting myself in bad situations while drinking and the day after”* – Male, 58, Sweden.

*“When you think you can drive despite drinking”* – Female, 64, Sweden.

### Intrusive thoughts

Whilst preceding themes had a consensus from both samples, Swedish participants highlighted a unique perception, not reported by British participants. They expressed how having pervasive thoughts regarding alcohol was inherently risky. In this instance personal perceptions are informed by experiential judgements using meta-cognitive reflection, in which the interruption of normal cognitive processes consequent of alcohol consumption is perceived as an indicator of risk:

*“When drinking takes up a large or significant part of daily thoughts”* – Male, 72.

*“Not being able to stop drinking*,* focusing on alcohol in all situations”* – Female, 48.

*“Thinking about the next time to drink”* – Female, 74.

The Swedish sample further expanded this sentiment and highlighted how alcohol interrupts cognitive processes through impacting inhibitory control and influencing subjective motivation to engage with alcohol consumption, such as the desire to drink:

*“When you feel you can’t stop and have a craving every day”* – Male, 64.

*“Hard to take a break from alcohol for example 1 month or so. The dose of alcohol gradually increases without you thinking about it”* – Male, 70.

*“When you feel that you have to take one not because you like the taste”* – Male, 46.

In forming these risk perceptions, experiential judgements are attuned by the affective reactions to the perceived vulnerability of developing problems with alcohol. This suggests an underlying concern or worry over problem drinking may influence self-reflection processes for risk perception. This sentiment was explicitly stated by one participant:

*“Fear of becoming an alcoholic”* - Female, 72.

### Personal relevance of risky drinking guidelines

Responses to this question suggest that risk guidance is not interpreted as intended, with participants expressing affective responses to the guidelines in terms of scepticism and denial - those that exceed the limits do not perceive their behaviour as risky. In contrast individuals who interpret the guidance in terms of personal risk, also experienced an affective reaction, expressing ambivalence over reducing their consumption. This highlights incongruity between personal perceptions of risk and the guidelines, but also the current risk guidance may be limited for facilitating behaviour change.

### Scepticism

A recurrent theme in both samples was scepticism towards national guidelines. Participants evaluated the guidance using experiential judgements, informed by their subjective tolerance for alcohol, their normative beliefs regarding peers’ consumption and situational drinking contexts. This resulted in affective responses of distrust or emotional reactance, in which they refuted the guidelines as too stringent - noting they did not align with their lifestyle, social behaviours, personal experiences, or account for individual differences:

*“Exaggerated. I’ve been drinking the same way since my teens”* – Male, 57, Sweden.

*“The limits feel quite low*,* most people I know drink more than 10 drinks a week”* - Male, 63, UK.

*“Wise but not realistic*,* I can drink 14 units in one go!”* – Male, 59, UK.*“I think four per occasion is a little low for a party or dinner”* – Female 59, Sweden.

*“I drink more and believe that these limits don’t apply to everyone”* – Male, 36, Sweden.

This highlights that participants did not process the guidelines in the manner they were intended, i.e., evaluating their own consumption and consequent risk in relation to the low-risk thresholds. This suggests that the risk information is not processed as intended by health authorities; guidelines were not internalised in terms of personal risk, hence motivation to drink within the limits may be low.

Furthermore, perceptions of unrealistically low limits highlight a cultural disconnect with drinking cultures in which heavy alcohol consumption is a normalised practice. The Swedish sample extended this perception by highlighting a sense of cultural relativism in which they perceived the guidelines as overtly strict in comparison to other nations. This again highlights that guidance is not interpreted as intended but rather with the use of experiential judgements:

*“The guidelines are arbitrary compared to different countries”* – Male, 53.

*“Have many European friends—Italy*,* France*,* Spain*,* Portugal—who find the Swedish attitude toward alcohol amusing. Wine with lunch isn’t unusual there”* – Male, 68.

Nonetheless, whilst the majority of both samples assessed relevance of the guidance using experiential processes, perceiving the guidelines did not reflect their personal experiences, a proportion (*n* = 3) of the Swedish sample did use logical processing to interpret the guidelines. These participants highlighted the subjective worth of the recommendations for informing their drinking behaviour:

*“I am aware of this and often reflect on it*,* evaluating my drinking”* – Female, 48.

*“I drink less than the limit values*,* but they help me keep my consumption down”* – Female, 64.

*“I think I’m going to cut down even more”* – Male, 55.

These sentiments suggest that guidelines *can* guide drinking decisions and highlight that these messages are internalised as intended by some.

### Denial and subjectivity

In addition to being sceptical, both samples downplayed the risk of exceeding the guidelines and perceived minimal concern over the potential consequences:

*“I drink too much but don’t think it’s dangerous”* – Male, 64, UK.

*“Overconsumption according to accepted norms*,* but I do not consider it risky”* – Male, 59, Sweden.

*“I feel that my alcohol consumption is not risk-filled even after several drinks”* – Male, 67, Sweden.

*“Far above* (the guidelines), *and it has been like that for over 40 years. I’m still not an alcoholic”* – Male, 66, Sweden.

Participants here evaluated the guidelines using experiential processes, in which they appraised personal risk on retrospection of their past drinking and resultant outcomes. This highlights use of a heuristic in which their subjective consequence-free experiences render the guidelines as personally irrelevant. Nonetheless, recalling only consequence-free drinking episodes suggests a potential rosy-retrospection (i.e., a bias for positive memories). This is reflected in the affective response to the guidelines - participants display emotional distancing, in which they recognise their drinking habits could be harmful, and perhaps are for other people, but not acknowledging it as a serious issue for them.

The experiential judgements and resultant affective responses may have been driven by the perception of being able to control their drinking despite regularly exceeding the limit. Using this reasoning to vindicate risky drinking behaviour may reflect cognitive dissonance, in which the discomfort from the risk is downplayed by rationalising they can control their drinking:

*“I drink what I want*,* and when I want it*,* no issues”* – Male, 66, UK.

*“Pretty high*,* borderline but under control”* – Male, 50, Sweden.

*“I drink far over the recommended intake but still feel in control”* – Male, 59, Sweden.

Whilst most participants evaluated the guidance using experiential and affective means, a portion of the British sample engaged with logical processes, in which they assessed their current drinking habits in relation to the guidelines. However, these evaluations were based on their subjective assessment of successfully reducing their intake, rather than drinking within or below the risk-threshold:

*“I probably drink/drank more than the recommended allowance but have reduced my alcohol intake considerably over the last two months”* – Male, 50.

*“I have substantially reduced my drinking*,* but I still drink double the guidelines”* – Male, 59.

*“I used to drink way over the guidelines but now I drink less”* – Female, 65.

This highlights that when individuals consider the guidance in terms of making informed decisions regarding alcohol-use, they subjectively judge their risk levels using personal experiences with drinking, rather than the objective information on risk espoused by the guidelines.

### Ambivalence

Whilst many participants were sceptical about the guidelines, or in denial over the risk of exceeding them, a portion of both samples acknowledged the risk but expressed ambivalence about reducing their consumption. In evaluating the guidance these participants engaged with deliberative judgements, rationalising consuming too much alcohol and expressing a desire for change. This highlights that for some the guidelines are interpreted in terms of personal risk and can potentially influence decision-making. Nonetheless, these evaluations gave rise to affective responses, in which they conveyed a struggle to implement the changes needed, highlighting an internal conflict and a sense of ambivalence:

*“Would like to reduce consumption or quit drinking altogether. Feel that I drink a little too often and occasionally too much”* – Female, 54, Sweden.

*“Tried to live by the earlier limits*,* but with the new ones*,* my drinking is unfortunately even more off”* – Male, 73, Sweden.

*“I think I drink too much when I do*,* and too many times a week. I want to cut back*,* but it’s hard”* – Female, 65, UK.

Further perceptions from the Swedish sample highlight some potential factors that may be driving the internal conflict for change. Engaging with experiential judgements, these participants were able to highlight this struggle may be result of the impairing effect of alcohol on decision-making, and contextual factors they link to heavy drinking. This highlights that making informed decisions regarding alcohol intake and risk is more nuanced than the current focus of quantity and frequency of drinking:

*“After one glass of wine*,* you often want another*,* and then you’ve lost track and end up having one or two more. It doesn’t feel good*,* and I don’t feel good about it.*

*Then I’ve passed the limit”* – Female, 67.

*“I drink too much when I socialise with certain people. Want to avoid that”* – Female, 51.

*“I often drink more during vacations or social events”* – Female, 55.

## Discussion

The current study used TA, embedded in a framework analysis, to explore risky drinking perceptions and personal relevance of drinking guidelines in a sample of Swedish and British drinkers seeking support to reduce their alcohol consumption. Results suggest risky drinking perceptions are formed using experiential and affective judgements and relate to the concerns of developing alcohol use disorders, a loss of control, drinking to cope, and a worry of experiencing personal harm or causing harm to others. In terms of personal relevance of the guidelines, individuals mainly interpreted them with experiential judgments, resulting in cognitive-affective responses of scepticism and denial – exceeding the guidelines was seen by some as inconsequential. Individuals who interpreted guidelines in terms of personal risk also expressed a cognitive-affective reaction – ambivalence, in which they acknowledged the risk but conveyed a struggle to drink within the limits.

Both sets of participants used experiential judgements to form perceptions, often using drinking pattern recognition attuned by anticipated affect to heighten the sense of risk. The use of experiential learning to inform risk appraisal has been demonstrated previously, with individuals forming perceptions using experiences of negative or positive consequences [21], their subjective alcohol tolerance and drinking frequency [22, 23] and alcohol expectancies [24, 25]. Furthermore, individual perception of risk using heuristics derived from experience and affect have been demonstrated to have a greater impact on drinking intentions than those derived from deliberative and numerical estimates of risk [34]. The current results support this, participants typically referred to heuristics derived from experiential-affective cues rather than deliberative analysis, and this pattern recognition was a key driver of their risk appraisal. This sentiment is qualified by evidence showing that recognition of individual drinking patterns results in changes in perceived vulnerability [35].

Generally, perceptions across the two nations were similar, except for two major differences. First, the British sample highlighted subjective thresholds of certain types of alcohol as indicative of risk, which were much higher than national guidelines. This perception was not considered by the Swedes. This reflects a cultural difference in drinking styles, supported by evidence showing the UK has a higher rate of heavy episodic drinking than Sweden [2]. Nonetheless, it may also reflect the impact of each country’s alcohol policies - Sweden has a stricter policy for pricing and availability than the UK, for example higher unit prices and no marketing campaigns, such as sale promotions. Research modelling the impact of relaxing Sweden’s alcohol governance highlights that consumption rates would increase considerably [36] – meaning the policy measures in place are likely to result in less heavy episodic drinking, suggesting this behaviour may not be as normalised as it is in the UK, hence accounting for the contrast in perceptions.

Secondly, Swedish participants highlighted how changes in cognitive processes, in which thoughts about alcohol become invasive, subjective motivation to drink increases and a reduction of inhibitory control is indicative of risk - this perception was not expressed by the British participants. This finding may reflect differing individual-level understanding of risk, consequent of varying approaches to alcohol-risk communication between the nations. However, the finding is likely the result of a greater recruitment of Swedish drinkers compared to British. The perceptions expressed by the Swedes are in line with cognitive processing models of addiction, which highlight dependent or problematic drinking is associated with a variety of changes in cognitive processes [37], for example an elevated subjective desire or craving for alcohol [38]. Furthermore, they noted that a lack of inhibitory control was a risk factor, again this reflects current understandings of alcohol use disorders, in which individuals have difficulty in inhibiting impulses and/or reward driven behaviour [39, 40]. In reflecting current understandings of addiction, this lay perception of risk would likely also be perceived in a larger sample of British drinkers.

The current findings provide examples of how experiential and affect laden heuristics are used to interpret deliberative risk information – suggesting that for some the guidelines are not used as intended by the health authorities that create them. The findings also suggest that guidelines can be effective in the sense of priming some individuals for change, i.e., make them consciously aware of unhealthy drinking behaviours. Nonetheless, the expressed ambivalence for change exemplifies the nuance of the decision to reduce consumption, a barrier that cannot be addressed by the current guidelines. This provides an important implication for policy and practice; discussions about the guidelines and consumption levels should be contextualised in the motivations and meaning of drinking to help people understand their personal risk in context of population risk models. For example, while all individuals benefit from reducing their alcohol consumption, indicators including drinking alcohol alone, for self-medication, or if drinking impacts personal or professional life, could be used to give nuance to quantitative guidelines. Clearer communication of the links between drinking within guidelines and lessened risk are likely needed in health communications to promote awareness of this link. Recent updates to the Canadian guidelines may provide an example of how to achieve this; Canadians are now provided with a continuum of risk, in lieu of fixed amounts, that highlights that no amount of alcohol is completely risk-free [41]. To further explore these issues, the perceptions of healthcare professionals is needed. In doing so will enable a consensus between practitioners and the public as to what message should be promoted and how guidelines should be used.

Whilst the study provides insight into personal perceptions of risks, how they are formed, along with elucidating processes used to interpret risk guidance, there are study limitations. The nature of the data should be considered, for example the responses to the questions were typically short statements, ranging from single words to a few sentences. This does present potential issues for informational power (i.e., the appropriateness of the data in providing sufficient and relevant information to address the research questions [42]). Nonetheless, the study was able to recruit over 300 participants, ensuring a wide range of perspectives were captured. Additionally, other aspects of the study highlight the sufficiency for addressing the research questions; the study aims were narrow and focused, the majority of the sample were risky drinkers (i.e., exceeded the guidelines), hence they were well placed to provide insight into risky drinking and the risk guidelines, the analysis was theory driven (i.e., risk perceptions) and finally the analysis strategy was appropriate - a cross-case analysis searching for patterns in the data [42].

Generalisability may be an issue as most of the participants were middle-aged and had signed up to participate in a trial estimating the effectiveness of alcohol intervention content. Recruitment for the trial was aimed at individuals contemplating behaviour change, and whilst the perceptions reflect those who drinking excessively (i.e., exceeding the guidelines), they may not reflect individuals who have no intention to change behaviour. Future research assessing perceptions of these individuals is needed, especially as they are most likely the hardest to reach group in terms of reducing personal harms. Nonetheless, the study does provide a unique insight into the perceptions of a group that is understudied in the literature, who have increased their consumption over the last decades and are the group most likely to exceed the recommendations [9, 10].

Personal perceptions of risky drinking were primarily formed using experiential and affective judgements, which highlight a set of concerns pertaining to development of AUDs, losing control, drinking for self-medication purposes, and worries about experiencing personal and social consequences. The guidelines were interpreted using experiential judgments, resulting in affective responses that were expressed as negative attitudes towards the guidance. Individuals who acknowledge the risks express a struggle or ambivalence for change. Perceptions of risk extend beyond the current focus of frequency and quantity of consumption, whilst the guidance is not interpreted as intended, suggesting current guidelines may have limited utility for informing drinking decisions. In revising guidelines, communication efforts may need to balance epidemiological risks with personal perceptions of risk.

## Supplementary Information

Supplementary Material 1.

Supplementary Material 2.

## Data Availability

Data is not available as participants did not provide consent for their responses to be shared publicly. Contact joel.crawford@liu.se for further information.

## Footnotes

1 Pre-2016 UK guidelines, 3–4 units per day for men and 2–3 units per day for women.
