# Supplementary material for: Personal perceptions of risky drinking and alcohol guidelines – a qualitative analysis
Source: BMC Public Health. 2025 Sep 16;25:3049. doi: 10.1186/s12889-025-24296-6 (PMC12439381; doi:10.1186/s12889-025-24296-6)
Supplement: Supplementary file 1 — Supplementary Material 1. [file 12889_2025_24296_MOESM1_ESM.pdf]

Appendix B – Questionnaire items

“In the box below please describe your personal definition of ‘risky drinking?”

Swedish Sample

“The National board of Health and Welfare defines risky drinking as consuming 10 or more standard glasses per week or consuming 4 or more standard glasses per session, once a month or more (see below image for guidance)”.

Standard glass Medium-strength beer 50cl	Standard glass Strong beer 50cl	Standard glass Wine 10cl	Standard glass Fortified wine 6cl	Standard glass Spirits 4cl

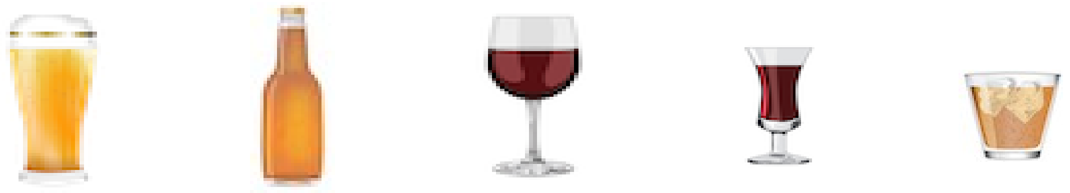

A standard glass contains 12g of pure alcohol

“How relevant is this guideline to you?”

British Sample

"The Chief Medical Officer defines risky drinking as consuming 14 or more units of alcohol per week (see below image for guidance)".

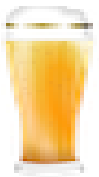	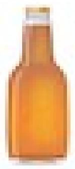	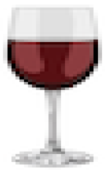	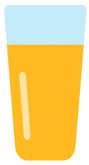	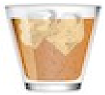	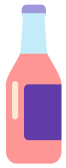
Pint of beer (4%) = 2.3 unit	Bottle of beer 330ml (5%) = 1.7 units	Glass of wine 175ml (12%) = 2.3 units	Pint of cider (4.5%) = 2.6 units	Shot of spirits 35ml (40%) = 1.4 units	Alcopop 275ml (4%) = 1.1 units

"How relevant is this guideline to you?"
